# Bradycardia, Hypotension, and Cardiac Arrest: A Complication of Local Anesthetics

**DOI:** 10.7759/cureus.4033

**Published:** 2019-02-07

**Authors:** Cynthia Balasanmugam, Carlos Henriquez Felipe, Daniel Rodriguez, Guy Kulbak

**Affiliations:** 1 Internal Medicine, Maimonides Medical Center, Brooklyn, USA; 2 Cardiology, Maimonides Medical Center, Brooklyn, USA

**Keywords:** local anesthetic toxicity, cardiotoxicity, bradycardia, sinus node arrest

## Abstract

Local anesthetics are routinely used in the field of medicine during many procedures, to alleviate acute pain. Although they are relatively safe, they have the ability to produce undesirable neurotoxic and cardiotoxic symptoms when administered intravascularly. Examples of cardiotoxicity include myocardial depression, cardiac arrhythmias, and cardiovascular collapse. The case below describes the adverse events of severe bradycardia, PR prolongation with subsequent heart block, and sinus arrest following an elective knee replacement in a 73-year-old male who received bupivacaine and ropivacaine.

## Introduction

The use of local anesthetics has been well-established in the field of medicine and is routinely administered by the majority of anesthesiologists and surgeons. These medications are relatively safe within their recommended dosage, but on rare occasion, can lead to undesirable systemic toxicities when introduced intravascularly. Cardiotoxicity, including myocardial depression and ventricular arrhythmias, have been infrequently identified with the use of local anesthetics but have critical adverse effects when encountered [1]. This case describes a 73-year-old male who was administered a local anesthetic during an elective total knee replacement procedure. Postoperatively, he was noted to develop symptomatic sinus bradycardia with PR segment prolongation, hypotension, and cardiac arrest.

## Case presentation

**Figure 1:**
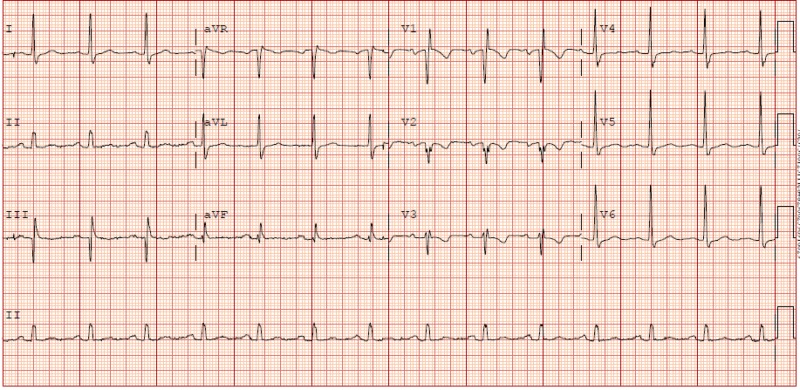
Preoperative EKG showing NSR with first-degree AVB, incomplete RBBB, septal Q waves EKG: electrocardiogram; NSR: normal sinus rhythm; AVB: atrioventricular block; RBBB: right bundle branch block

**Figure 2:**
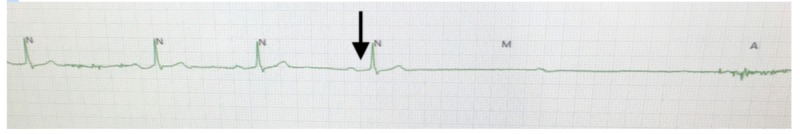
Postoperative PR prolongation (arrow) increased from baseline EKG with sinus bradycardia and eventually sinus arrest leading to asystole EKG: electrocardiogram

The patient was a 73-year-old man who was initially admitted for an elective right-sided total knee replacement to alleviate many years of suffering from severe osteoarthritis. His past medical history consisted of coronary artery disease, which led to a coronary artery bypass graft (CABG) procedure that was completed six years prior to this admission. The patient's baseline electrocardiogram (EKG) showed a normal sinus rhythm (NSR) with a first-degree atrioventricular (AV) node block and an incomplete right bundle branch block (RBBB) (Figure 1). His most recent echocardiography revealed an ejection fraction of 41%-45% with mild aortic valve stenosis. The surgery was completed without any complications. However, the patient received spinal anesthesia with bupivacaine preoperatively, as well as a right femoral nerve block with ropivacaine postoperatively. Subsequently, he was noted to have three episodes of bradycardia, hypotension, and one incident of cardiac arrest following the procedure. Upon a review of the telemetry strip, the patient developed severe sinus bradycardia with progressive sinus slowing to a heart rate in the 20s, PR interval prolongation, followed by a brief period of asystole (Figure 2). At the time of cardiac arrest, chest compressions were started, atropine was administered, and return of spontaneous circulation (ROSC) was achieved within 20 seconds. A transvenous pacer was placed and the patient was treated for suspected local anesthetic toxicity in the postanesthesia care unit (PACU) with Intralipid, with a resolution of symptoms. The patient’s heart rate and blood pressure returned to baseline following the post-infusion of Intralipid. He was then evaluated by electrophysiology (EP) and a loop recorder was placed without any evidence of a complete heart block or a high degree of atrioventricular (AV) nodal block.

## Discussion

Local anesthetics are used extensively for a wide array of procedures in order to control acute pain. Amine-based anesthetics, such as bupivacaine, levobupivacaine, and ropivacaine, are commonly administered to control these acute pains at the rare expense of developing cardiac toxicity.

The toxicity of these drugs has been associated with the high plasma concentrations of these anesthetics, initially showing signs of central nervous system toxicity. This includes lightheadedness, dizziness, and even loss of consciousness. Cardiovascular adverse effects can be a sign of severe systemic toxicity, with early excitatory effects causing tachycardia and hypertension, followed by cardiovascular collapse [1].

The most dangerous acute cardiac effects are seen following unintended intravascular injections of anesthetics. Local anesthetic agents work by preventing the conduction of nerve impulses primarily in nerve cell membranes by the inhibition of voltage-gated Na+ channels, slowing the conduction of the action potential and altering the membrane's threshold [2]. This can lead to a change in the excitability of cardiac cells, including the firing rate of the sinoatrial node, which, in turn, can have unfavorable effects on heart rate and blood pressure. EKG findings indicated that the effects were most pronounced in the case of bupivacaine with a widening of the QRS complex and prolonged PR intervals [3-4]. At increasing doses, bupivacaine and levobupivacaine were also found to be associated with diminished cardiac contractility in animal models, with bupivacaine, in particular, showing a significant reduction in ejection fraction [5].

## Conclusions

In the current case, telemetry monitoring was an extremely valuable tool in identifying the sudden decompensation of this patient and allowed us to commence the advanced cardiac life support (ACLS) protocol immediately, with a favorable outcome. Upon review of the telemetry strip, we were able to quickly identify changes from the patient's baseline EKG, including periods of severe bradycardia with a gradual slowing of the heart rate, PR prolongation, eventual heart block, and sinus arrest.

Since the patient had no prior history of pre-syncope, syncope, dizziness, or lightheadedness, it gave us reason to suspect his symptoms were consistent with rare local anesthetic toxicity. For further evaluation, a loop recorder was placed and carefully reviewed, which did not identify any baseline abnormalities in his heart rhythms, including complete heart block, a high degree AV block, or ischemic changes. The resolution^ ^of his symptoms with an Intralipid infusion also made a vagally mediated response less likely.

In order to decrease the rate of systemic toxicity as well as cardiac adverse events, there are many safety steps that can be completed while administering a local anesthetic. Telemetry monitoring postoperatively or post the administration of a local anesthetic can help us quickly identify an assess systemic or cardiac toxicity in a timely fashion.
